# Whole-genome sequence of the strictly anaerobic bacterial strain SANA belonging to the family *Gottschalkiaceae*, isolated from a xenic culture of an anaerobic protist

**DOI:** 10.1128/mra.00174-24

**Published:** 2024-05-20

**Authors:** Ryuji Kondo, Takafumi Kataoka

**Affiliations:** 1 Department of Marine Science and Technology, Fukui Prefectural University, Obama, Fukui, Japan; California State University San Marcos, San Marcos, California, USA

**Keywords:** whole-genome sequence, strictly anaerobic bacterium, *Gottschalkiaceae*

## Abstract

An anaerobic bacterial strain SANA was isolated from a xenic culture of an anaerobic heterolobosean protist which was obtained from a saline lake in Japan. Its draft genome comprises 1 circular chromosome (3,490,293 bp), harboring 3,275 predicted protein-coding and 73 tRNA-encoding genes and 8 rRNA operons.

## ANNOUNCEMENT

An anaerobic bacterial strain SANA was isolated from a xenic culture of a heterolobosean protist established via enrichment of bottom sediment from the saline Lake Hiruga (35°36.212′N, 135°53.349′E) in Fukui, Japan, in December 2015, by using a modified DSMZ 195c medium (https://www.dsmz.de/microorganisms/medium/pdf/DSMZ_Medium195c.pdf) (1) with Bacto Tryptone (1.0 g/L) and heat-inactivated horse serum (Gibco) (10 mL/L) substituted for Na-L-lactate and without selenite-tungstate solution in solution A (hereafter RYUJi’s marine medium). An aliquot of the protist culture was inoculated in the RYUJi’s marine medium and incubated at 20°C in the dark. After several transfers to the same medium, a single colony was isolated using the anaerobic agar-shake tube technique (2) in November 2019. A BLAST search (3) for strain SANA 16S rRNA gene sequence, obtained by direct sequencing of PCR amplicon using a bacterial universal primer set (4), returned *Andreesenia angusta* strain MK-1^T^ (NR_044642) and *Gottschalkia acidiurici* strain 9a^T^ (CP003326) with sequence identities of 89.6% and 89.0%, respectively. This suggested that strain SANA represented a novel genus and species of the family *Gottschalkiaceae* according to species delineation using 16S rRNA gene sequence (5). The genome sequence of strain SANA is a valuable resource to define the relatedness among species in the family *Gottschalkiaceae*, which consist of only three species of *Gottschalkia purinilytica*, *G. acidiurici,* and *A. angusta* (6).

Strain SANA was grown at 20°C in a 1-L anaerobic culture bottle (Sanshin) containing 500 mL RYUJI’s marine medium. Then, the cell pellet was harvested by centrifugation at 3,310 × *g* and 4°C for 30 min. Genomic DNA (gDNA) was extracted from the cell pellet by the standard phenol-chloroform method, as described previously (7), and then purified using Short Read Eliminator XS (Circulomics). A long-read DNA library of 10–20 kb-sized fragment prepared using g-TUBE (Covaris) was constructed using the SMRTbell Express template prep kit v2.0 (PacBio) and sequenced using the PacBio Sequel IIe system. High-fidelity (Hi-Fi) reads were obtained from the subreads generated using circular consensus sequencing via SMRT Link v10.1.00.119528. After shearing the gDNA into 500 bp, meanwhile, short-read DNA library was prepared by MGIEasy PCR-Free DNA Library Prep and sequenced using DNBSEQ-G400 (PE200, MGI). The 33,834 Hi-Fi reads were corrected by comparing with the short-read using Filtlong v0.2.1 (https://github.com/rrwick/Filtlong) to screen more than 5,000 bp. The corrected 22,645 reads were assembled using the Flye v2.9 (8) with auto min-overlap and assembly type of meta. Default parameters were used for all software unless otherwise specified. This resulted in a single 3,490,293 bp circular genome with a 54 × coverage (*N*_50_ = 3,490,293 bp) and an overall G+C content of 42.1% as determined using DFAST (9). The circular contig was rotated to the *dnaA* gene comes first using DFAST with “fix_origin option” (9).

The draft genome annotated using DFAST (9) contained 8 ribosomal RNA operons, 74 tRNA genes, and a total of 3,726 coding DNA sequences including 1,434 genes encoding hypothetical proteins. All eight 16S rRNA gene sequences were found to be identical by multiple alignment analysis of 16S rRNA genes.

## Data Availability

The genome sequence of the SANA has been deposited in DDBJ under the accession number AP028876. The raw reads have been deposited in the DDBJ Sequence Read Archive under accession numbers DRR498520 and DRR498521. The BioProject and BioSample accession numbers are PRJDB16408 and SAMD00636377, respectively.
